# 

**Published:** 1911-08-19

**Authors:** 


### GIFTS.

The late Mr. Arthur Smith, of Leicester, has bequeathed £100 to the Leicester Infirmary.

Mr. Benjamin Bamber Gardner, M.A., of Lancaster, formerly a solicitor, has left £100 to the Lancaster Infirmary.

Mrs. Catherine Pigott has left £100 each to the Surgical Aid Society, and to the Middlesex Hospital for the cancer ward.

Mr. Frederick Scorer, of Edenbridge, Kent, has left £50 each to the Hospital for Children, Great Ormond Street, and to the Free Cancer Hospital, Fulham.

The Grocers' Company has given £20 to the After Care Association for Poor Persons discharged recovered from Asylums for the Insane, Church House, Westminster, S.W.

In response to the appeal of the Hon. Harry Lawson, M.P., for the funds of the Royal Hospital for Incurables, Putney Heath, "A Friend" has sent a donation of a thousand guineas.

The late Mrs. Wright, of King Street, Potton, Beds, a life governor and annual subscriber for many years, has bequeathed one-twelfth of the residue of her estate to Addenbrooke's Hospital, which it is expected may amount to about £1,000.

Miss Jane Elizabeth Cunningham, of Finsbury Park, has left £25 per annum each to the New Hospital for Women and the Hospital for Incurables at Putney, and she directed that any surplus income from her estate should be paid in equal shares to these and one other institution.

Mr. Henry Charles Lane, managing director of the Nugget Polish Company, Ltd., has bequeathed £500 to the Belgrave Hospital for Children, Clapham Road, to endow a "Harry Lane Cot"; and £200, free of all duties, to the Maternity Home, Chantilly, France; also £500 to his medical man, Dr. George Nicol Henry.

### SEPTEMBER CHANCE FOR A SHIP'S DOCTOR.

The Peninsular and Oriental Company announce a cruise of nineteen days by their new "durbar" steamer *Maloja* (12,500 tons), from London, on September 23, to Lisbon, Teneriffe, Madeira and Gibraltar, at fares of sixteen to thirty guineas. The *Vectis* is to be despatched from London on September 9 for a month's cruise to the Dalmatian Coast and Venice, and in October to Constantinople and Greece. An illustrated programme of these cruises may be had at the Company's offices.

### BUILDINGS CONTEMPLATED AND IN PROGRESS.

Lewisham.—Mr. A. Griffin, F.S.I., who was architect for the original buildings of St. John's Hospital, on Morden Hill, Lewisham, is designing the extension, which is to be a memorial to the late King. Messrs. Parker, of Peckham, are the builders. New bacteriological and *x*-ray departments, together with an enlargement of the male ward, is the extent of the addition.

Merthyr Tydfil.—Mr. Thomas Roderick is architect for the proposed sanatorium at Pontsarn. Tenders are invited for foundation contract and also for extensive railings and gates.

Norman's Riding, Durham.—It is proposed to build a porter's lodge at the Isolation Hospital, Norman's Riding. Tenders are to be addressed to the Clerk, Council Offices, Blaydon-on-Tyne.

Norwich.—Mr. R. I. Green, of Norwich, is architect for the infirmary, which is to contain 175 beds.

Nottingham.—Extensions are contemplated to the Children's Home, Edward Lane. Mr. W. B. Starr, of Nottingham, is the architect.

Nuneaton.—Mr. E. E. Shepherd, M.S.D., of Nuneaton, is architect for the proposed Children's Home at Nuneaton. Fifteen tenders have been received, the highest £1,750 and the lowest £1,240, by Mr. E. Fox, of Leicester.

Oakwell (Yorks).—A Local Government Board inquiry has been held regarding an application of the Oakwell Joint Hospital Board for sanction to borrow £4,000 to pay for hospital extensions. Evidence was given by the architect, Mr. Burrows.

Portsmouth.—The Guardians contemplate an extension of the infirmary, and tenders amounting to twenty-one in number have been received. The lowest, by Mr. Cockerell, of Southsea, is £39,989, the highest £51,883.

### JOTTINGS.

Hope Hospital, at Fort Wayne, Ind., U.S.A., is to be rebuilt at a cost of $100,000. The present quarters are to be abandoned.

The Women's Hospital, Philadelphia, is to be provided with a roof-garden in commemoration of its recently celebrated fiftieth anniversary.

An investigation into the management of the tuberculosis sanatorium at Mount Vernon, Ohio, which was built at a cost of $600,000, has disclosed the fact that for forty-eight patients in the institution, no fewer than fifty-three attendants had been provided, costing the State $50,000 annually for maintenance.

The scheme for the establishment of a School of Tropical Medicine in Calcutta is now being matured. It is understood that the cost of the institution will be borne jointly by the Bengal and Imperial Governments. Meanwhile, the Bengal Government has been asked to work out details of the school and to submit plans and estimates. It is hoped that the school will be ready for a post-graduate course to be given to medical men in India when the cool weather starts.

### OBITUARY.

Professor Charpy, of Toulouse, an anatomist well known for his work on the morphology of the thorax and pelvis, died recently in that town from a chill following the ingestion of ice.

James O'Hara Scanlon, Lieutenant-Colonel of the retired Army Medical Staff, has died at Broadclyst, Devon, at the age of sixty-four. He was a graduate of the University of Glasgow, and took degrees of M.B. and C.M. in 1868.

### APPOINTMENTS VACANT.

Full particulars of these vacancies can be obtained on reference to our advertisement columns:

Buxton, Devonshire Hospital.—Assistant House Surgeon.
Cumberland Infirmary, Carlisle.—Resident Medical Officer.
Glasgow Western Infirmary.—Lecturer on Clinical Methods.
Liverpool Royal Southern Hospital.—House Surgeons and House Physicians.
Manchester Children's Hospital.—Visiting Surgeon and Male Resident Medical Officer.
Queen's Hospital for Children, Hackney Road.—House Surgeon.
Royal Albert Hospital, Devonport.—Assistant House Surgeon.
Royal Surrey County Hospital, Guildford.—House Surgeon, Assistant House Surgeon.
Sunderland Royal Infirmary.—House Physician.

### TO HOSPITAL SECRETARIES AND OTHERS.

We invite contributions from any of our readers, and shall be glad to pay, according to length, for items of news for the institutional section (including appointments, resignations, gifts, etc.) which we may publish. All payments for manuscripts used are made as early as possible after the beginning of each quarter.

